# Fabrication and Application of a Microfluidic Chip for Biofilm Cultivation and Analysis under Controlled Flow

**DOI:** 10.1021/acsomega.5c04643

**Published:** 2025-08-13

**Authors:** Adei Abouhagger, Eivydas Andriukonis, Goda Grigorianaite, Raiane Rodrigues da Silva, Kamile Kasperaviciute, Arunas Stirke, Wanessa C. MA Melo

**Affiliations:** Department of Functional Materials and Electronics, 226274 State Research Institute Centre for Physical Sciences and Technology (FTMC), 02300 Vilnius, Lithuania

## Abstract

Microbial biofilms present significant challenges in healthcare due to their persistence and resistance to antimicrobial treatments. Microfluidic technologies offer a promising alternative to traditional static systems for studying biofilm dynamics under physiologically relevant conditions. In this study, we present a poly­(dimethylsiloxane) (PDMS)-free microfluidic platform fabricated using off-stoichiometry thiol–ene (OSTE) resin and cyclic olefin copolymer (COC) substrates. The device features five independent growth chambers and is designed for compatibility with standard laboratory setups. It enables controlled flow conditions, optical transparency for real-time imaging, and integration with antimicrobial testing protocols. Biofilms of *Staphylococcus aureus* and *Pseudomonas aeruginosa* were cultivated under dynamic flow and compared to static cultures in tissue culture wells. Confocal microscopy was used to assess structural features, viability, and thickness over time. The dynamic environment supported more uniform and spatially organized biofilm growth, while static conditions led to denser but structurally heterogeneous formations. Treatment with different tetracycline concentrations demonstrated effective biofilm disruption, particularly under flow, confirming the platform’s utility for evaluating antimicrobial efficacy. With a fabrication cost below five dollars per chip and potential for cleaning and reuse, the platform offers a cost-effective and scalable solution for biofilm research. This study highlights the advantages of OSTE–COC microfluidics in modeling biofilm-associated infections and provides a practical tool for real-time biofilm analysis and therapeutic screening.

## Graphical abstract

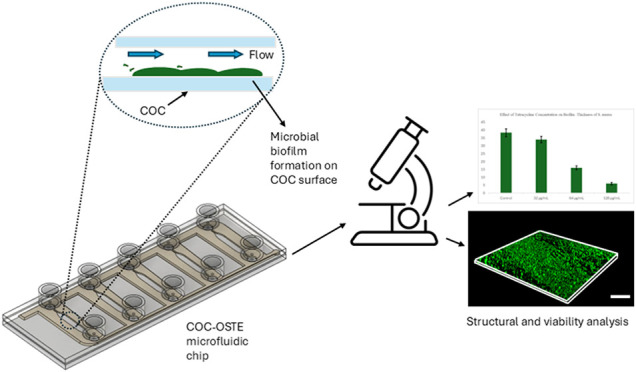

## 1 Introduction

Microbial biofilms are structured communities of microorganisms embedded within a self-produced extracellular polymeric matrix (EPS), which protects them from environmental stressors such as immune responses, antibiotics, and dehydration.^1,2^ This resilience contributes to their role in persistent infections, particularly those associated with chronic wounds, indwelling medical devices, and hospital-acquired infections—over 75% of which involve biofilms .^3^ Understanding biofilm development and resistance mechanisms is essential for advancing infection control strategies, yet conventional static culture models often fail to replicate the dynamic and complex environments in which biofilms naturally form .^4^

Microfluidic technologies have emerged as powerful tools for biofilm research, offering precise control over flow conditions, nutrient gradients, and shear forces to better simulate physiologically relevant conditions .^5^ Compared to traditional static systems, microfluidic platforms enable high-resolution, real-time observation of biofilm formation, structure, and response to treatment .^6–8^ Their miniaturized design also allows for efficient reagent use and integration with analytical techniques, making them ideal for investigating dynamic biofilm behavior.

PDMS-based microfluidics are widely used in microfluidic chip fabrication, with studies like Zhang et al. demonstrating the importance of controlled flow in mimicking physiological conditions and assessing antimicrobial efficacy.^9^ Straub et al. explored biofilm formation and antimicrobial treatment using a PDMS-based microfluidic platform, emphasizing real-time analysis under controlled conditions.^10^ Similarly, Tang et al.^11^ developed a microfluidic chip to study the dynamics of antibiotic resistance in bacterial biofilms, showing that subinhibitory ciprofloxacin concentrations could select for resistant mutants, highlighting the utility of microfluidics in resistance evolution research. Hhowever, PDMS presents notable drawbacks, including absorption of small molecules, deformation under pressure, and surface hydrophobicity ,^12–14^ which can interfere with reproducibility and limit experimental fidelity.

Alternative materials, including cyclic olefin copolymer (COC) and off-stoichiometry thiol–ene (OSTE) polymers, have recently gained attention in biofilm research as promising solutions to address the material-related limitations of PDMS. COC offers advantages including low small-molecule absorption, high optical clarity, and mechanical robustness. Cesaria et al. demonstrated that COC can modulate bacterial colonization, showing distinctive biofilm morphology and reduced biomass adhesion compared to traditional substrates such as PDMS.^15^ Additionally, OSTE materials have been evaluated for their suitability in supporting *Staphylococcus aureus* biofilms within microfluidic environments. Amorim et al. conducted a systematic study characterizing OSTE polymer surfaces for *S. aureus* biofilm cultivation, highlighting their compatibility with biofilm formation and their potential for rapid microfluidic prototyping.^16^ These recent studies underscore the growing interest in PDMS alternatives for biofilm research platforms.

In this study, we developed a poly­(dimethylsiloxane) (PDMS)-free microfluidic chip designed for biofilm cultivation and analysis under controlled flow conditions. The chip features five independent growth chambers and is fabricated using off-stoichiometry thiol–ene (OSTE) resin and cyclic olefin copolymer (COC) substrates. This material combination offers several advantages over conventional microfluidic platforms, including enhanced durability, reduced costs, and compatibility with standard laboratory equipment. Additionally, the chip incorporates standardized slide dimensions and Luer ports, allowing for flexible experimental setups, such as imaging, antimicrobial treatment testing, and potential integration with biosensors.

Using this platform, we investigated the growth dynamics of biofilms formed by *Pseudomonas aeruginosa* and *S. aureus* under dynamic flow conditions. The results were compared to traditional static systems to evaluate the chip’s capability in replicating physiologically relevant environments. Furthermore, biofilm structural parameters, including thickness, surface coverage, and viability, were analyzed under both control and antibiotic treatment conditions. This study highlights the potential of microfluidic systems for advancing biofilm research, offering a robust and cost-effective platform for studying biofilm-associated infections and exploring effective therapeutic strategies.

## 2 Materials and Methods

### 2.1 Materials and Reagents

COC TOPAS microscopy slide format (75.5 mm × 25.5 mm) and a microscopy slide platform with 2 × 5 Luer connectors were procured from Microfluidic ChipShop, Jena, Germany. Sylgard 184 silicone elastomer kit was supplied by Dow Corning. The PDMS master mold was fabricated using Zortrax white resin on a Zortrax Inkspire three-dimensional (3D) printer (Olsztyn, Poland). OSTE resin (Ostemer 322) was obtained from Mercene Laboratories, Stockholm, Sweden. PTFE tubing with a 1/32″ inner diameter, used for media delivery, was purchased from Darwin Microfluidics, while Luer adapters were sourced from Microfluidic ChipShop, Germany. Brain Heart Infusion (BHI) broth was acquired from Biolab, Hungary. Ciprofloxacin was obtained from Thermo Fisher Scientific. For visualization, MycoLight bacterial viability assay kit (AAT Bioquest) was used.

### 2.2 Microfluidic Chip Fabrication

The microfluidic chip is composed of two COC substrates, one of which is a Microscopy slide format (bottom COC) and the other features a microscopy slide Luer platform with ten fluidic interfaces (top COC) (Microfluidic ChipShop, Jena, Germany). The top COC was modified by drilling precise holes at the Luer connection centers to enable fluid passage into the microchannels. The drilled holes were aligned to ensure unobstructed flow and proper integration with the underlying microfluidic system. The COCs were precleaned with acetone, rinsed with isopropanol, and cleaned in an ultrasonic bath for 10 min. Afterward, the slides were treated with oxygen plasma (Zepto B, Diener Electronic, Germany) at 50% power and 0.35 mbar pressure for 90 s. The PDMS mold was aligned with the bottom COC slide, and OSTE resin (Ostemer 322, Mercene Laboratories, Stockholm, Sweden) was injected into the cavity via PTFE tubing connected to injection ports. The fabrication process is illustrated in Figure 1.

**Figure 1:**
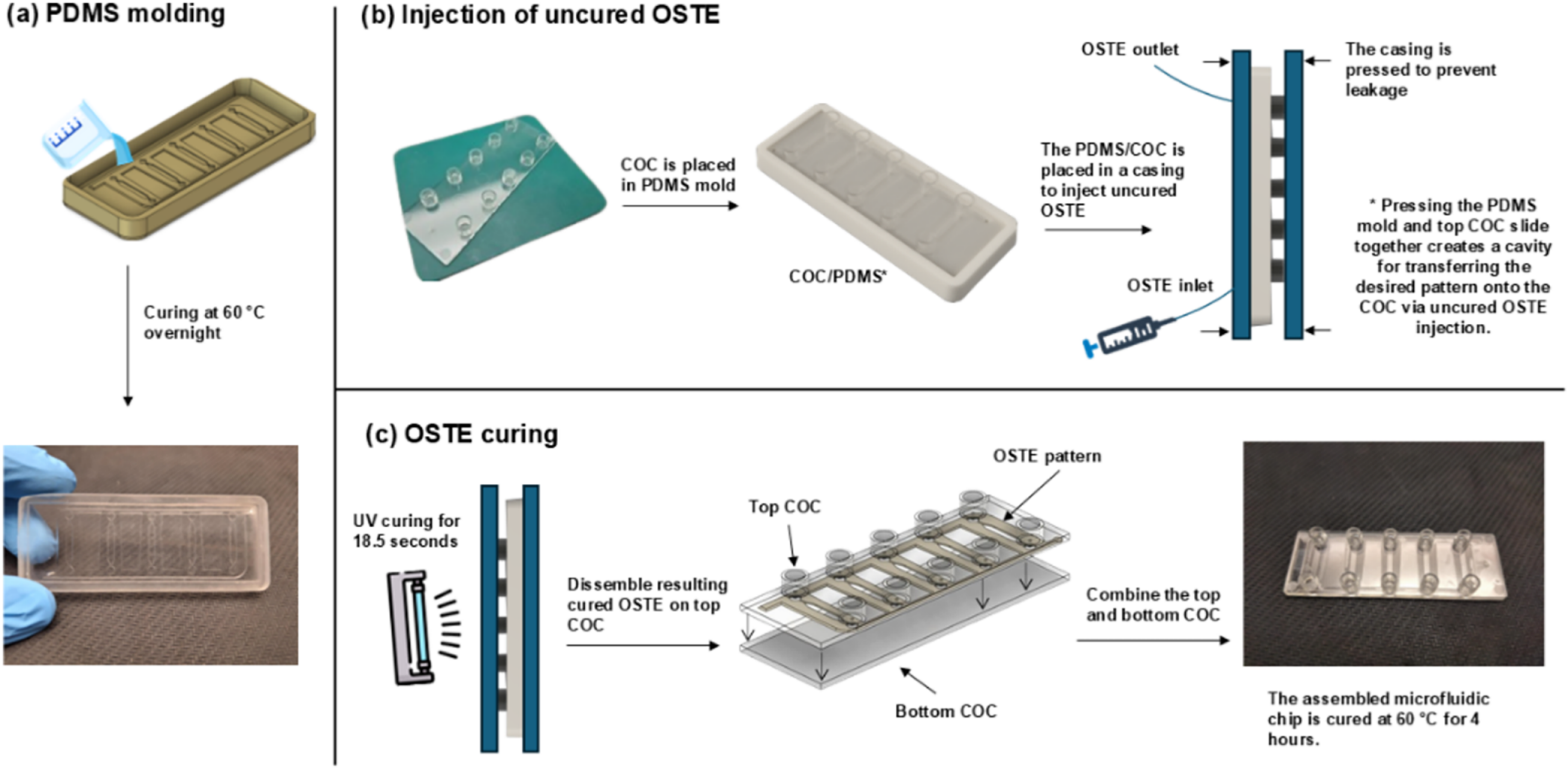
Overview of the microfluidic chip fabrication process. The process involves (a) PDMS mold preparation, (b) OSTE resin injection, and (**c**) UV curing and final assembly of the COC slides.

The OSTE resin was prepared by mixing components (Part A(1.09): Part B(1.0), w/w), degassed, and injected into the cavity. Initial curing on the top COC was performed with 365 nm UV light at an intensity of ∼2.04 mW/cm^2^ for 18.5 s. As a result, a layer of sticky OSTE was depicted on the top COC featuring the structures of the microchannels. Next, the bottom COC was aligned with the sticky OSTE layer and the microchannels were sandwiched and sealed between them. The microfluidic chip was additionally cured at 60 °C for 4 h to achieve complete OSTE polymerization, facilitate bonding, and ensure proper sealing of the microchannels. The resulting microfluidic chip consisted of two COC plates forming the top and bottom channel surfaces, with OSTE polymer exclusively constituting the channel sidewalls. The microfluidic chip was disinfected with 70% isopropanol, inspected for potential leakage, and the channel dimensions were measured using a Vernier calliper to verify accuracy. It was then stored in a sterile Petri dish for subsequent experimentation.

### 2.3 Microbial Biofilm Cultivation

#### 2.3.1 Cultivation in the Microfluidic Chip

*S. aureus* (ATCC 25923) and *P. aeruginosa* (PAO1) were grown aerobically in BHI at 37 °C and 150 rpm, until mid logarithmic phase. Then, the culture was centrifuged at 5000 rpm for 5 min and washed twice with phosphate-buffered saline (PBS). The cell suspension was standardized with a spectrophotometer adjusted at 620 nm wavelength to a final concentration of 1 × 10^7^ cells mL^–1^. Subsequently, the microchannels were filled with the standardized cell suspension using a micropipette and it was incubated at 37 °C under static conditions for 1.5 h to facilitate initial cell adhesion to the surface.

The microfluidic system was assembled and prepared for experimentation. Within the chip, bacterial cells adhered to and grew on the COC surfaces lining the microchannels during biofilm cultivation. The microfluidic chip was connected to a flow control system using sterile tubing and Luer connectors to ensure a sealed, contamination-free setup. A syringe pump (Model ISPLab02, InfuseTek, China) was calibrated to deliver precise flow rates and operated under controlled conditions to simulate the desired shear stress within the microchannels. Following initial bacterial cells adhesion, the microfluidic chip was connected to syringes containing fresh BHI growth media, which was perfused through the channels at a flow rate of 5 μL/min for the duration specified by the experimental protocol. Following the growth phase, PBS was perfused through the microfluidic chip at the same flow rate of 5 μL/min for 30 min to remove residual waste and nonadherent material, preparing the chip for further experimentation and staining. All experiments were conducted in at least three biological replicates. The five growth chambers integrated into the microfluidic chip were used simultaneously, either to replicate conditions within a single experiment or to compare different treatment conditions under identical flow parameters.

#### 2.3.2 Biofilm Cultivation in Cell Culture Plate

Sterile tissue culture plates were prepared by placing a 1 cm × 1 cm COC square into each well to match the chip material, followed by the addition of 1 mL of standardized bacterial suspension (1 × 10^7^ cells/mL). The plates were incubated at 37 °C under static conditions for 1.5 h to allow initial bacterial cell adhesion. Following this, the wells were gently washed three times with sterile PBS to remove nonadherent cells. Subsequently, 2 mL of BHI broth was added to each well, and the plates were incubated at 37 °C on an orbital shaker set to 50 rpm to promote biofilm formation for the duration specified by the experimental protocol. After incubation, planktonic cells were carefully removed by aspirating the supernatant, followed by two gentle washes with 1 mL of sterile PBS to remove residual debris while preserving the adhered biofilm. The prepared biofilms were then used for further analyses, including staining, imaging, and viability assessments.

### 2.4 Biofilm Treatment

Mature *S. aureus* biofilms grown for 48 h were treated with tetracycline hydrochloride at concentrations of 32, 64, and 128 μg/mL, delivered in PBS for 16 h. During the treatment, flow was maintained at 5 μL/min to ensure continuous exposure of the biofilm to the antibiotic solution. After treatment, the channels were flushed with sterile PBS at the same flow rate for 30 min to remove residual antibiotic and loosely attached cells.

### 2.5 Biofilm Staining

#### 2.5.1 Staining for the Microfluidic Chip

The cultivated microbial biofilm in the microfluidic chip were stained using the MycoLight bacterial viability assay kit, which employs a dual-staining method with MycoLight Green and propidium iodide to distinguish live and dead cells based on membrane integrity. A working solution of the dye was prepared by mixing equal volumes of MycoLight Green and propidium iodide to create a 250× stock solution, according to the manufacturer’s instructions. The biofilms were gently rinsed with PBS to remove residual media and stained by introducing the working solution into the microfluidic channels. The staining solution was incubated at room temperature for 25 min in the dark. Excess stain was subsequently removed by perfusing PBS through the channels at a flow rate of 5 μL/min for 10 min prior to imaging.

#### 2.5.2 Staining for the 24-Well Plates

The microbial biofilms grown in 24-well plates were also stained using the MycoLight bacterial viability assay kit. After removing the media and washing the wells three times with PBS to remove planktonic cells, 100 μL of the working dye solution was added to each well. The plates were incubated at room temperature for 25 min in the dark to allow staining. Excess stain was removed by gently washing the wells twice with PBS to ensure removal of unbound dye, and the biofilms formed on the COC were prepared for imaging.

### 2.6 Confocal Microscopy and Biofilm Characterization

Stained biofilms were visualized using a Nikon Eclipse Ti2 confocal laser scanning microscope equipped with a 20× objective lens. Fluorescence was detected at an excitation wavelength of 488 nm, with emission filters set to 510–530 nm for MycoLight Green (live cells) and 600–660 nm for propidium iodide (dead cells). Images were captured using an Andor Zyla sCMOS camera integrated with the DSD2 differential spinning disc system, providing high-resolution and precise fluorescence detection. Z-stack images were acquired at 1 μm intervals to enable three-dimensional reconstruction and z projection of the biofilm architecture.

Image acquisition was performed using Nikon Elements software, and subsequent analysis was conducted in ImageJ (Fiji). Z-stack slices were compiled into volumetric renderings, allowing for visualization of biofilm structure and surface coverage. Biofilm thickness was quantified by identifying the first and last slices exhibiting fluorescence above background levels along the *z*-axis, with thickness calculated based on the total number of slices and the z-step size.

## 3 Results

### 3.1 Microfluidic Chip Fabrication

A microfluidic platform featuring five identical growth chambers was designed to facilitate biofilm cultivation and treatment under controlled flow conditions. Each chamber allows for independent experiment conditions while maintaining uniform fluid dynamics across the system. The chip fabrication process combined photolithography and soft lithography techniques, utilizing a 3D-printed positive master mold and OSTE resin to create precise and mechanically robust microchannel structures. Assembly involved bonding the patterned OSTE layer to a COC substrate using plasma treatment, ensuring strong adhesion and channel integrity. Leak testing confirmed the reliability of the bonding process, with no fluid leakage observed across a range of tested flow rates. The chip demonstrated excellent reproducibility, with channel dimensions varying by less than ±2% across multiple fabrication iterations. With standard microscope slide dimensions (75 mm × 25 mm), the platform offers a compact, imaging-compatible format suitable for diverse biofilm research applications. Overall, this microfluidic system provides a reliable and versatile tool for investigating biofilm formation, structure, and response to treatment under dynamic flow conditions.

### 3.3 Comparative Analysis of Biofilm Development (Static vs Dynamic Method)

The progression of biofilm growth for *P. aeruginosa* and *S. aureus* was monitored at 3, 24, and 48 h postcell adhesion under both dynamic (microfluidic chip) and static (COC pieces in 24-well plate) conditions (Figure 2). These time points were chosen to capture critical stages of biofilm development: the early adhesion phase (3 h), the maturation phase (24 h), and the establishment of a mature biofilm (48 h).

**Figure 2:**
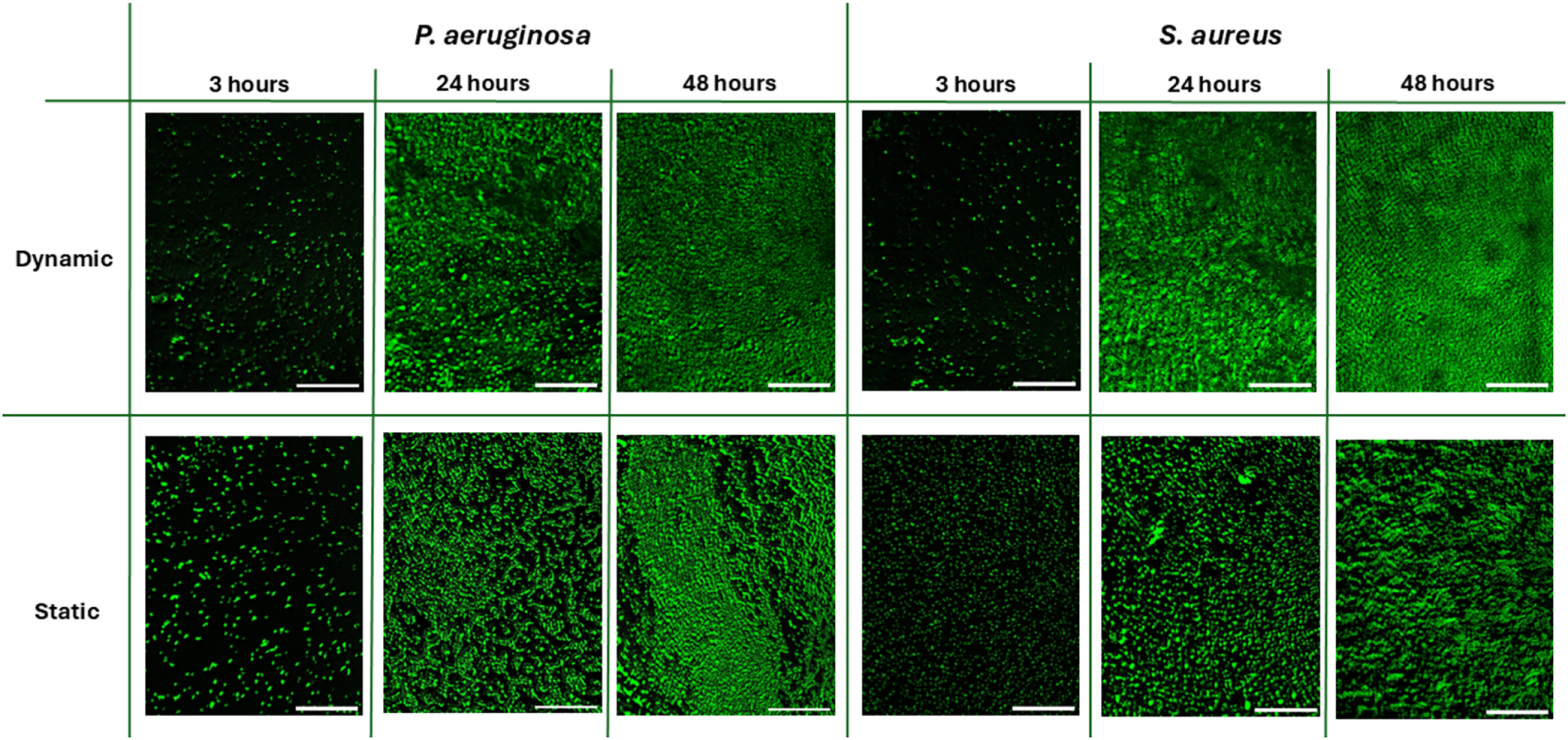
Confocal fluorescence images of *P. aeruginosa* and *S. aureus* biofilms cultivated under dynamic and static conditions at 3, 24, and 48 h postadhesion. Maximum intensity projections of z-stacks were used to visualize overall biofilm coverage. Top: dynamic conditions. Bottom: static conditions. The results presented in this figure are representative of the same experiment repeated at least three times, producing similar results every time. Scale bar: 100 μm.

At the 3 h time point, both *S. aureus* and *P. aeruginosa* exhibited sparse surface attachment under both static and dynamic conditions, consistent with the early stages of biofilm development and initial bacterial adhesion. By 24 h, a noticeable increase in fluorescence intensity was observed for both species, reflecting the establishment of early biofilm structures. After 48 h of incubation, both organisms formed denser, surface-associated biofilms with increased surface coverage.

Under dynamic conditions, *S. aureus* demonstrated a more uniform and continuous fluorescence signal at 48 h, indicative of well-distributed biofilm formation across the growth chamber. *P. aeruginosa* also formed continuous biofilms under flow, but the fluorescence signal varied across the surface, indicating uneven biofilm thickness or density. In static conditions, both species showed progressive biomass accumulation over time, with *P. aeruginosa* displaying a tendency toward localized clustering. The images are representative of at least three independent experiments and illustrate consistent patterns of biofilm growth over time. These results support the platform’s capability to facilitate and monitor reproducible biofilm development under controlled dynamic conditions.

To further visualize the vertical architecture of biofilms formed under different cultivation conditions, 3D reconstructions were generated from confocal z-stacks acquired after 48 h (Figure 3). Both *S. aureus* and *P. aeruginosa* developed biofilms with multilayered structures under static and dynamic conditions. Quantitative analysis of z-stack data revealed that *S. aureus* biofilms reached an average thickness of 38.3  ±  2.49 μm under dynamic flow, while *P. aeruginosa* biofilms exhibited a lower average thickness of 27.3  ±  1.92 μm.

**Figure 3:**
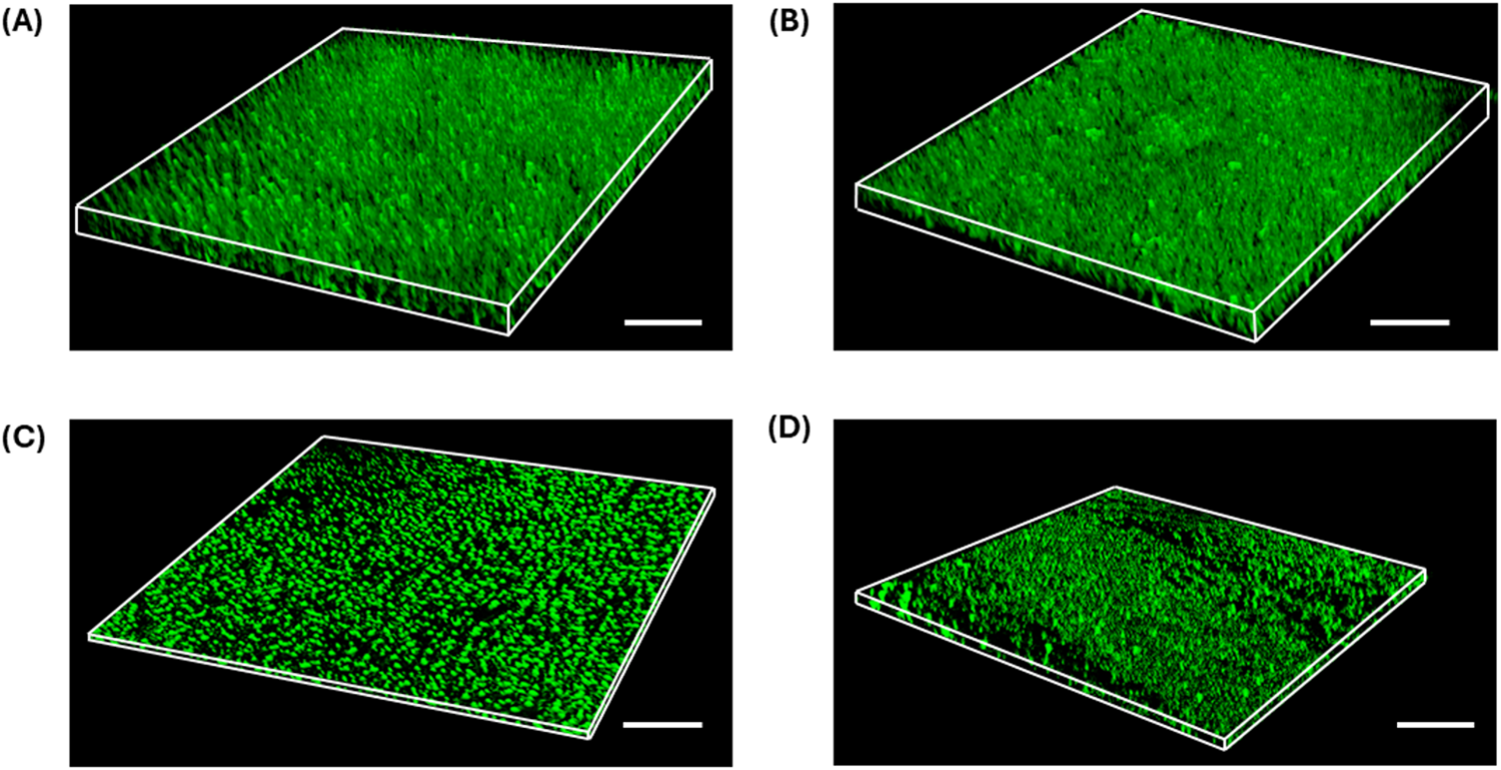
Three-dimensional reconstructions of *S. aureus* and *P. aeruginosa* biofilms after 48 h of cultivation under dynamic and static conditions. Confocal z-stacks were acquired at 1.0 μm intervals and rendered into volumetric views. Panel (A) shows *S. aureus* under dynamic flow, and panel (B) shows *P. aeruginosa* under dynamic flow. Panels (C, D) show *S. aureus* and *P. aeruginosa* biofilms formed under static conditions, respectively. Each image is representative of at least three independent experiments. Scale bars: 100 μm.

### 3.4 Effects of Antibacterial Treatment on Biofilm Architecture

The effects of antibacterial treatment on biofilm structure and viability were evaluated for *S. aureus* biofilms grown under dynamic conditions for 48 h. The biofilms were stained to distinguish live cells (green fluorescence) from dead cells (red fluorescence), and structural changes were assessed through CLSM imaging and biofilm thickness measurements (Figure 4).

**Figure 4:**
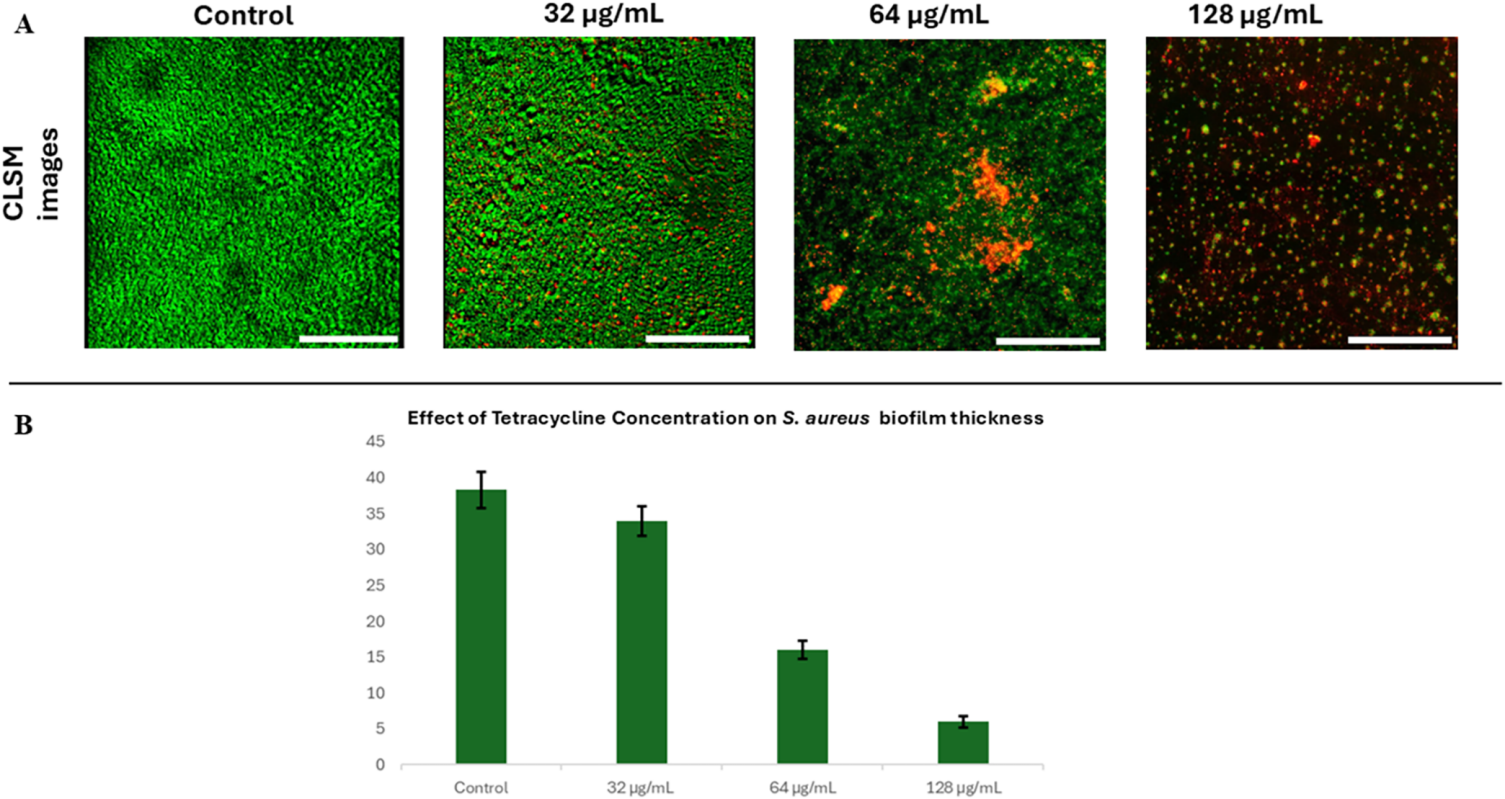
Effect of tetracycline on *S. aureus* biofilms cultivated in the microfluidic chip. (A) CLSM images of *S. aureus* biofilms after 48 h growth followed by 16 h treatment with increasing concentrations of tetracycline (32, 64, and 128 μg/mL). Biofilms were stained using the MycoLigh Bacterial Viability Kit. A dose-dependent increase in red signal and reduction in biomass is observed. Scale bar = 100 μm. (B) Quantification of biofilm thickness following treatment, based on z-stack reconstructions. Data represent mean ± standard deviation from three independent experiments.

CLSM revealed a concentration-dependent effect of *S. aureus* biofilms following tetracycline treatment. In untreated control samples, biofilms appeared dense and uniformly distributed, exhibiting predominantly green fluorescence indicative of high cell viability. Exposure to 32 μg/mL tetracycline resulted in a largely preserved biofilm structure; however, a modest increase in red fluorescence, primarily localized to the upper biofilm layers, suggested the initial onset of bacterial cell death. At 64 μg/mL, the biofilm exhibited visible gaps, structural discontinuity, and increased red signal, reflecting both cell death and biomass reduction. The most pronounced change occurred at 128 μg/mL, where biofilm coverage was sparse and red fluorescence dominated, consistent with extensive cell death and possible detachment of the nonviable biomass.

Quantitative analysis of biofilm thickness supported these observations, with average thickness decreasing from 38.3 μm in controls to 34.0, 16.2, and 6.3 μm at 32, 64, and 128 μg/mL, respectively. While part of the observed biomass loss may result from antibiotic-induced detachment of nonviable cells, the combined reduction in biofilm thickness and altered structural appearance indicates a substantial impact on biofilm integrity.

## 4 Discussion

PDMS has long been the material of choice in microfluidic device fabrication due to its ease of use and optical clarity. However, it presents several limitations, including the absorption of small molecules such as antibacterial agents,^12^ deformation under pressure,^13^ and hydrophobicity,^14^ which may influence biofilm behavior and experimental outcomes. For instance, Recent findings have shown that PDMS retains significant amounts of lipophilic compounds due to bulk absorption, resulting in delayed washout and increased variability, whereas COC exhibits minimal sorption—primarily through reversible surface interactions—providing more reliable performance in applications involving drug exposure and bacterial cultures.^17^ In contrast, OSTE polymers have been shown to exhibit superior chemical resistance, structural robustness, and dimensional precision,^18–22^ making them particularly suitable for applications that demand stable and consistent microfluidic performance, such as biofilm cultivation and analysis.

In this study, we present a PDMS-free microfluidic platform designed to address key limitations commonly encountered in conventional biofilm research. Unlike standard PDMS-based devices, the system is primarily constructed from COC, with OSTE used for forming the channel sidewalls. This material combination offers enhanced chemical resistance, mechanical durability, and dimensional precision, supporting experimental reproducibility. The chip incorporates five independent growth chambers and conforms to standard microscope slide dimensions, allowing seamless integration with conventional laboratory equipment. Its optical transparency enables real-time visualization of biofilm development and fluid dynamics. The fabrication process is cost-effective, with an estimated material cost of under five dollars per chip following master mold preparation. Additionally, the use of reusable molds, low-cost materials, and rapid UV-curing steps renders the method amenable to scale-up without the need for cleanroom facilities or specialized infrastructure. Owing to the chemical robustness and stable bonding of the OSTE–COC construction, the chip can be reliably cleaned and reused across multiple experimental cycles without compromising structural integrity. Channels can be sterilized using enzymatic or chemical agents, supporting repeated biofilm cultivation and treatment studies at low cost. These features establish the platform as a practical, scalable, and sustainable alternative to PDMS-based systems for dynamic biofilm research and antimicrobial testing.

The comparative analysis of biofilm development under flow and static conditions highlights the critical influence of fluid dynamics on biofilm morphology and organization. Within the microfluidic system, the presence of continuous shear forces appeared to constrain vertical biomass accumulation and support the formation of more spatially uniform and structurally coherent biofilms. In contrast, static conditions lacked this regulatory effect, leading to uncontrolled biomass growth and heterogeneous architecture, particularly evident in *S. aureus* cultures. These findings are consistent with prior reports that associate shear stress with the suppression of excessive matrix accumulation and improved surface-level organization.^23,24^

The dynamic behavior observed in our platform is more representative of clinical environments, where biofilms commonly develop under flow conditions such as those present in urinary catheters, vascular devices, and endotracheal tubes. In such settings, fluid shear plays a key role in shaping bacterial adhesion, biofilm structure, and treatment response. For instance, Gomes et al. highlight that hydrodynamic forces substantially influence biofilm formation on indwelling medical devices, impacting both colonization and biofilm stability.^25^ Tsagkari et al. demonstrate that complex shear dynamics significantly affect the development and spatial organization of multispecies biofilms, reinforcing the importance of modeling physiologically relevant flow conditions .^26^ Similarly, De Grazia et al. report that bacterial attachment is reduced in high-shear regions of microfluidic models simulating ureteral stents, further supporting the relevance of shear-controlled environments for studying clinical biofilms.^27^ Together, these findings support the rationale for employing dynamic systems to better replicate the conditions under which biofilms form and persist *in vivo*.

The results of our study demonstrate that fluid flow conditions significantly influence the spatial organization, distribution, and morphology of developing biofilms. Under dynamic conditions, *S. aureus* exhibited a more uniform and continuous biofilm structure, while *P. aeruginosa* biofilms were thinner and less aggregated compared to static conditions (Figure 3), where both species developed thicker and more heterogeneous structures. These findings align with previous research highlighting that shear forces can suppress excessive vertical biomass accumulation and enhance planar biofilm organization.^24^ Notably, Zhang et al. demonstrated that hydrodynamic shear stress led to thinner, denser *P. aeruginosa* biofilms with more homogeneous surfaces and altered live-cell distribution profiles, indicating improved nutrient transport and metabolic gradients.^28^ Their study also reported that biofilms formed under flow were significantly less permeable and exhibited greater resistance to antibiotics which further support the relevance of dynamic biofilm cultivation under physiologically realistic, shear-influenced environments such as those encountered on medical devices and in fluid-exposed tissues.

The results of tetracycline treatment demonstrated a clear concentration-dependent effect on *S. aureus* biofilms cultured within the microfluidic platform. At 32 μg/mL, the biofilm remained largely intact, with predominantly green fluorescence observed via confocal microscopy, suggesting that this dose functioned below the minimum biofilm inhibitory concentration (MBIC) threshold for mature *S. aureus* biofilms. This is consistent with previous findings indicating that subinhibitory concentrations of tetracycline may alter bacterial physiology without inducing significant cell death.^29^ In contrast, treatment with 64 μg/mL led to partial disruption of the biofilm structure and an increase in red fluorescence, indicative of membrane-compromised cells. The most substantial effects were observed at 128 μg/mL, where the biofilm exhibited a marked reduction in thickness and widespread red fluorescence, consistent with significant bacterial killing. These outcomes align with prior studies reporting MBEC values for doxycycline—an analogue of tetracycline—typically falling within the 64 to 128 μg/mL range for *S. aureus* biofilms.^30^ Moreover, the use of a microfluidic flow environment likely enhanced antibiotic penetration and uniform exposure, overcoming diffusion limitations common in static systems. This observation is consistent with findings by Tang et al., who demonstrated that continuous flow improved antimicrobial delivery and exposure uniformity in *Escherichia coli* biofilms, leading to more effective treatment outcomes compared to static cultures.^11^ Together, these findings highlight both the efficacy of high-dose tetracycline treatment and the value of the microfluidic chip in enabling realistic, high-resolution assessment of biofilm susceptibility under physiologically relevant conditions.

While this study focused on *P. aeruginosa* and *S. aureus* as model organisms, the approach is readily adaptable to other bacterial species, antimicrobial agents, and experimental configurations. The platform’s customizable design and optical clarity make it possible for future integration of components such as electrodes for real-time electrochemical monitoring or embedded sensors for microenvironmental control. Where metals can be patterned on COC^15^ using masks, prior to OSTE bonding and microchannel sealing. Its flexibility also supports the simulation of diverse physiological conditions, highlighting its potential as a valuable tool for advancing biofilm research and evaluating antimicrobial strategies.

## 5 Conclusions

This study presents a cost-effective, PDMS-free microfluidic platform for investigating microbial biofilm development and response to antimicrobial treatments under dynamic flow conditions. The chip is fabricated using COC, which serves as the main structural material and the surface on which biofilms grow within the microchannels. OSTE was used to form the channel sidewalls, providing dimensional precision and robust bonding. The platform overcomes key limitations of conventional PDMS-based systems by offering improved chemical resistance, mechanical durability, and compatibility with standard laboratory infrastructure.

Using *S. aureus* and *P. aeruginosa* as representative organisms, we showed that continuous flow supports the formation of structurally distinct biofilms compared to static conditions, with more uniform surface coverage and constrained biomass accumulation. Antibiotic treatment experiments with tetracycline further validated the platform’s ability to assess biofilm susceptibility under dynamic microenvironments, revealing a concentration-dependent reduction in viability and thickness. These results are consistent with previous reports and underscore the importance of shear-controlled environments in modeling clinically relevant biofilm behavior.

Future developments will include the integration of biosensors, electrochemical components, or coculture configurations to further expand its utility in studying microbial communities, antibiotic resistance, and treatment efficacy in complex settings.
